# Aridity Gradients Shape Intraspecific Variability of Morphological Traits in Native *Ceratonia siliqua* L. of Morocco

**DOI:** 10.3390/plants12193447

**Published:** 2023-09-30

**Authors:** Jalal Kassout, Younes Hmimsa, Salama El Fatehi, Khalil Kadaoui, Mhammad Houssni, Soufian Chakkour, Abdelouahab Sahli, Mohamad Ali El Chami, David Ariza-Mateos, Guillermo Palacios-Rodríguez, Rafael M. Navarro-Cerrillo, Mohamed Ater

**Affiliations:** 1 Regional Center of Agricultural Research of Marrakech, National Institute of Agricultural Research, Avenue Ennasr, P.O. Box 415, Rabat Principale, Rabat 10090, Morocco; 2 Laboratory of Applied Botany, Bio-Agrodiversity Team, Faculty of Sciences, University of Abdelmalek Essaâdi, Tétouan 93030, Morocco; y.hmimsa@uae.ac.ma (Y.H.); s.elfatehi@uae.ac.ma (S.E.F.); khalil.kadaoui@outlook.com (K.K.); mhammadhoussni@gmail.com (M.H.); chakkoursoufian@gmail.com (S.C.); sahliabdelouahab@gmail.com (A.S.); mohammed.ater@gmail.com (M.A.); 3 TEDAEEP Team Research, Polydisciplinary Faculty of Larache (FPL), University of Abdelmalek Essaâdi, P.O. Box 745, Larache 92000, Morocco; 4 Forestry Engineering Department, ERSAF Research Group RNM-360, University of Córdoba, 14014 Córdoba, Spain; ep2elchami@uco.es (M.A.E.C.); b42armad@uco.es (D.A.-M.); gplacios@uco.es (G.P.-R.); rmnavarro@uco.es (R.M.N.-C.)

**Keywords:** *Ceratonia siliqua* L., functional traits, intraspecific variation, aridity gradient, Morocco

## Abstract

The carob tree (*Ceratonia siliqua* L.) is a significant fruit tree in the Mediterranean region with cultural, biological, and ecological importance. Despite its importance, intraspecific trait variability (ITV) in carob trees has been largely overlooked in previous studies. Understanding ITV and its relationship with environmental conditions is crucial for conservation and breeding programs. In this study, we investigated the variability of carob pod and seed-related traits across different ecological scales in 25 studied populations in Morocco. Significant differences in morphological traits were observed between carob populations at various ecological levels, and pod-related traits exhibited greater variability than seed traits. Correlation analysis revealed strong associations between carob morphological traits and environmental conditions, with altitude and aridity index playing an influential role. The aridity gradient was strongly related to changes in pod size, seed number, and size, as well as seed yield. Our findings highlight an important ITV reaching 45% at the intra-population level, 36.5% at the inter-geographic level, and 30% at the inter-population level. Overall, this study contributes valuable insights into the ecology and adaptation of carob trees, emphasizing the importance of considering intraspecific variability when studying this remarkable species. This knowledge is critical for addressing the challenges posed by climate change and human activities on the long-term survival and ecological functioning of carob populations.

## 1. Introduction

Plants, through the process of natural selection, evolve to develop an optimal phenotype best suited for their specific environmental niche. These selection processes give rise to a range of adaptation strategies unique to each plant species [1]. These strategies are reflected in the variation of plant functional traits, which, in turn, are pivotal in shaping ecological processes. They directly dictate the dynamics of community composition and ecosystem functions [2]. In the Mediterranean region, climate change projections foresee a decrease in precipitation, accompanied by rising temperatures and an increase in extreme weather events [3]. These impending shifts in environmental conditions have the potential to exert profound impacts on natural ecosystems. Of particular concern is the significant threat posed to the growth and development of trees and forest ecosystems [4,5]. Yet, research has shown that Mediterranean plant species possess remarkable phenotypic plasticity and a capacity to adapt their traits to cope with these challenging environmental conditions [5,6,7,8]. In matter of fact, phenotypic plasticity stands out as a crucial mechanism enabling plants to adapt to the dynamic and swiftly shifting climatic conditions that characterize the contemporary environment [5,9]. This adaptation is essential because the relatively slower pace of evolutionary processes often fails to match the rapidity of climate change [5]. The manifestations of this plasticity are observable across several ecologically significant traits encompassing morphology, physiology, anatomy, and phenology [10]. For instance, the wild olive tree (*Olea europaea* subsp. *europaea* var. *sylvestris*) exhibits substantial variability in leaf and wood traits in response to environmental factors such as aridity [8,11]. Similarly, *Quercus ilex*, a Mediterranean evergreen species, demonstrates significant phenotypic plasticity in response to the varying environmental conditions prevalent in the Mediterranean region [12]. Interestingly, despite the importance of functional trait variation, fruit tree species, such as the iconic carob tree, have received comparatively less attention in such studies. This oversight is especially noteworthy given the carob tree’s unique ecological significance and its potential to provide valuable insights into how fruit trees adapt to changing environmental conditions.

The carob tree (*Ceratonia siliqua* L.) is one of the most iconic fruit trees of the Mediterranean area and a marker of its cultural, biological, and ecological diversity [13]. Its origins are rooted in a palaeobotanical history of several million years and are linked to the Mediterranean climate [14]. Indeed, its origins and domestication history have been largely the subject of much debate [15,16]. Previous archaeological, palaeobotanical, and historical findings have suggested a single eastern origin followed by human-driven dissemination to the western Mediterranean [15,16,17]. These claims were accepted due to the development of grafting in the eastern Mediterranean and its key role in the domestication process of fruit trees [18]. However, recent phylogeographic evidence suggests a strong west–east genetic structuring and the presence of multiple domestication centers from native populations all over the Mediterranean basin [19]. Hence, Baumel et al. [19] highlighted west-to-east expansions of carob from a single long-term refugium located in the foothills of the High Atlas Mountains of Morocco. Moreover, the authors showed the existence of multiple origins of domestication at the Mediterranean scale, which was also supported by the strong west–east divergence in wild carob populations [20]. In addition, the assessment of plant species associated with the carob tree at the Mediterranean scale reveals a clear differentiation between western and eastern plant communities [21]. Together, these findings highlight the unique status of the carob tree as a wild element of the Mediterranean flora and a fruit tree that contributes largely to the history of the Mediterranean region.

Nowadays, the carob tree is commonly found in Mediterranean countries such as Spain, Italy, Greece, Turkey, and Morocco [21,22], and has been introduced to various regions worldwide [13]. Its ability to thrive in dry and marginal areas [17], withstand environmental variability [22], and resist drought and salinity [23,24] has increased its socio-economic value as a multipurpose tree [13]. With a growing demand for carob-based products, there has been increased interest in its cultivation and propagation, leading to numerous studies on its ecology and distribution [13,21,22], phylogeny and evolution [13,22,25], morphology [26,27,28], and biochemistry of pods, seeds, and leaves [29,30,31,32,33]. Additionally, its high nutritional and bioactive components, such as phenols, flavonoids, vitamins, and minerals [29,34,35], make it an exceptional functional food [36,37]. However, with the current global changes and increasing anthropogenic pressures on natural habitats, the sustainability of the carob tree is under threat. Therefore, understanding the variability of pods and seeds in response to environmental conditions is crucial to developing new breeding programs and preserving genetic resources [22,38].

The study of intraspecific traits variability (ITV) in carob trees represents an emerging and crucial dimension in the broader field of plant biology. While research on various aspects of carob morphological traits has been abundant, ITV has remained relatively overlooked, although exceptions exist (as indicated by [22]). The prevalent approach in existing studies has been to assess trait variability by examining only a limited number of individuals from one or a few populations, subsequently averaging the results at the species level. Unfortunately, this narrow perspective has left significant gaps in our understanding of how carob trees respond to the diverse environmental gradients encountered across different ecological scales. As investigations have advanced, it has become evident that ITV constitutes a substantial and influential component of the overall variability in plant traits, substantiated by a growing body of research (e.g., [8,11,22,39,40]). This body of evidence underscores the pivotal role played by ITV in facilitating plant adaptation to an expansive spectrum of environmental conditions, encompassing diverse climatic, ecological, and geographic settings [22,39,40,41,42,43].

Against this backdrop, the present study is undertaken with two principal objectives. Firstly, we seek to conduct a meticulous and comprehensive examination of the extent, range, and structural patterns characterizing ITV in traits specifically related to carob pods and seeds. This endeavor is pivotal in shedding light on the intricacies of trait variation within carob populations, offering insights into the mechanisms underpinning carob’s remarkable adaptability. Secondly, we aim to delve into the impact of environmental factors, with a particular emphasis on aridity, in sculpting the observed variability in these traits. As aridity represents a significant environmental stressor in many carob-growing regions, understanding its role in shaping ITV not only enhances our comprehension of carob tree biology but also holds broader implications for addressing the challenges posed by changing environmental conditions. In essence, this study endeavors to unravel the hidden dimensions of ITV in carob trees, ultimately contributing to a more holistic and nuanced understanding of how these resilient plants navigate the complex interplay between their genetic makeup and the environmental forces that shape them.

## 2. Results

### 2.1. Variation in Carob Pod and Seed Traits between Populations

All of the studied traits showed highly significant differences between the 25-studied carob populations (Table 1). At the inter-population level, pods, and seed traits of Ceratonia siliqua showed substantial variation (Table 1). For instance, pod weight (PoWe) and seed weight (SeWe) showed a significant amplitude of variability with a coefficient of variation (CV%) of 45.03% and 35.11%, and an F-ratio of 211.55 and 110.82, respectively. The pod weight of carob populations ranged from 15.30 ± 3.83 g (Afertane population) to 3.98 ± 1.35 g (Ait Daoued population), and seed weight ranged from 2.58 ± 0.56 g (Afertane population) to 0.75 ± 0.33 g (Ait Daoued population). Seed yield also showed significant variability with a CV of 30.25% and an F-ratio of 99.26, with values ranging from 32.85 ± 6.46% (Arbaa Tigdouin population) to 16.15 ± 4.54% (Imi N’Tlit population). Seed length, width, and thickness displayed a CV of 10.57%, 10.74%, and 11.51%, respectively. Accordingly, seed length of carob populations ranged from 10.19 ± 0.76 mm (Iaasfa population) to 8.17 ± 0.84 mm (Komch population), seed width ranged from 7.66 ± 0.83 mm (Amtel population) to 6.17 ± 0.66 mm (Komch population), and seed thickness ranged from 4.72 ± 0.55 mm (Imheouach population) to 3.97 ± 0.31 mm (Ouaouizeght population). Generally, pod-related traits displayed larger variability than seed traits (Table 1).

Significant differences were found between the North, Center, and South regions when considering mean values of traits per geographic zone (Supplementary Table S1). Multiple comparison tests of Tukey HSD post hoc indicate that carob populations from the Center and South regions show similar variation in pod length (PoLe), pod central thickness (PoCT), aborted seed number (ASeN), and seed width (SeWi). Therefore, populations from the three geographic zones were different regarding pod width (PoWi), pod marginal thickness (PoMT), pod weight (PoWe), seed weight (SeWe), pulp weight (PuWe), seed yield (SeY), seed individual weight (SeIWe), seed length (SeLe), and seed thickness (SeT). However, populations from the North and Center regions were found to be similar for seed number (SeN).

The linear discriminant analysis showed that LD1 accounted for 85.5% of the total variance, and it was positively correlated with all traits, except for seed yield (SeY). Notably, carob trees from the North region were separated from those of the Center and South regions (Supplementary Figure S1). The correlation between geographic distance and trait variability across the studied populations resulted in a significant relationship (*p* < 0.001, RM = 0.339). However, when considering individual traits separately, non-significant correlations were found for PoLe, PoCT, SeY, SeLe, and SeT (Supplementary Table S2).

### 2.2. Structure and Amount of Intraspecific Variation in Carob Traits

**Figure 1:**
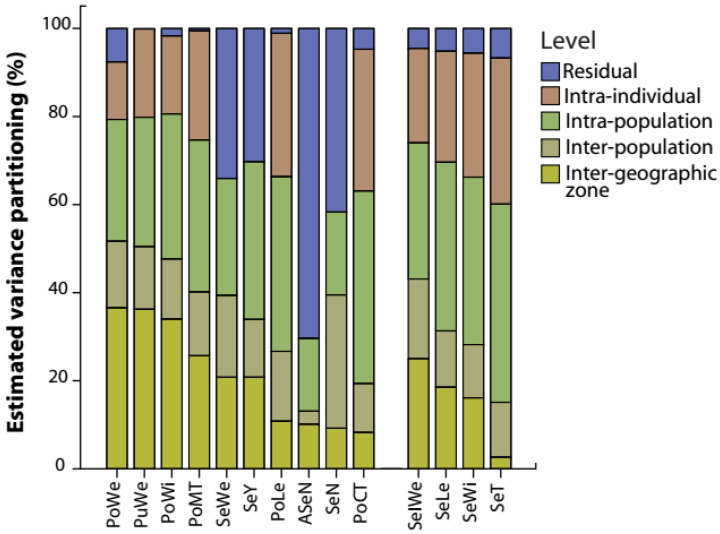
Estimated percentage variance across hierarchical levels and carob traits. Abbreviations for pod and seed traits are given in Table 1.

The variance decomposition of carob morphological traits revealed differences in the estimated portion of variance explained across the studied ecological scales from individual trees to geographic zones (Figure 1). Differences between individual trees of the same population captured from 16.50% to 45% of the total variability. Therefore, SeIWe, SeLe, SeWi, SeT, SeY, SeWe, PoCT, PoMT, and PoLe had a higher portion of variance at the intra-population level (ranging from 26.57% to 45%). PoWe, PuWe, and PoWi expressed a large portion of variance at the inter-geographic level, i.e., between geographic zones. PoLe and PoCT showed an important portion of variance explained by differences between pods of the same tree. Seed-related traits exhibited an important portion of variance between seeds of the same tree (Figure 1). Generally, except for SeN, a small fraction of variance was observed between populations (i.e., inter-population level) compared to other hierarchical levels (Figure 1).

**Figure 2:**
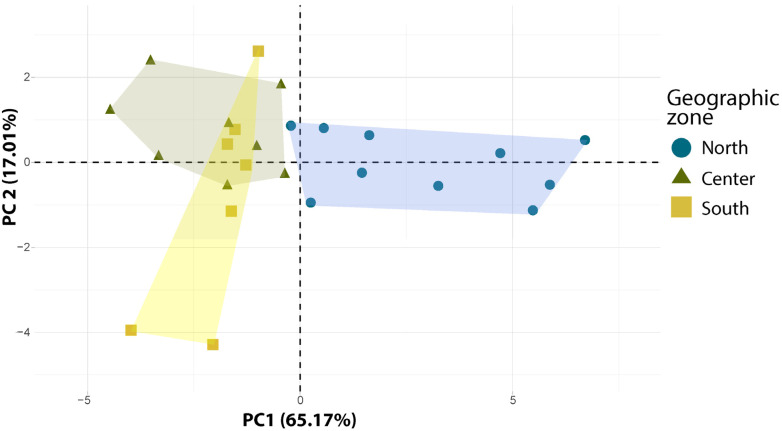
Principal component analysis (PCA) of the studied carob populations belonging to different geographic zones.

The principal component analysis (PCA) of pod and seed traits revealed that the first two axes explained 82.17% of the total variation (Figure 2, Supplementary Table S3). The first PCA axis (capturing 65.17% of the total variation) showed a significant and positive correlation with PoLe, PoWi, PoMT, PoCT, PoWe, SeWe, ASeN, SeIWe, SeLe, SeWi, and SeT, while exhibiting a significant and negative correlation with SeY. The second PCA axis (capturing 17.01% of the total variation) showed a significant and positive correlation with SeN, SeY, SeWe, and PoLe (Supplementary Table S3). The first PCA axis represented a discrimination axis of populations from the North region, loaded on the positive side of the axis, compared to those from the Center and South regions loaded on the negative side of the first PCA axis. This implies that populations from the North region have longer, wider, and thicker pods with a greater number of bigger and wider seeds compared to populations from the Center and South regions. Populations from the Center and South show a higher seed yield (SeY) compared to populations from the North. However, populations from the South region appear to be distributed along the second PCA axis (17.01%), which reflects differences in trait values, such as SeN, SeY, and SeWe, between populations located in the South and Center regions (Figure 2). The discrimination highlighted in the PCA analysis between populations from the North and those from the Center and South regions was confirmed by the hierarchical clustering analysis (Supplementary Figure S2). Populations from the North clustered into the first group and those from the Center and South in the second group (Supplementary Figure S2).

### 2.3. Carob Traits Variation According to Environmental Variables

**Figure 3:**
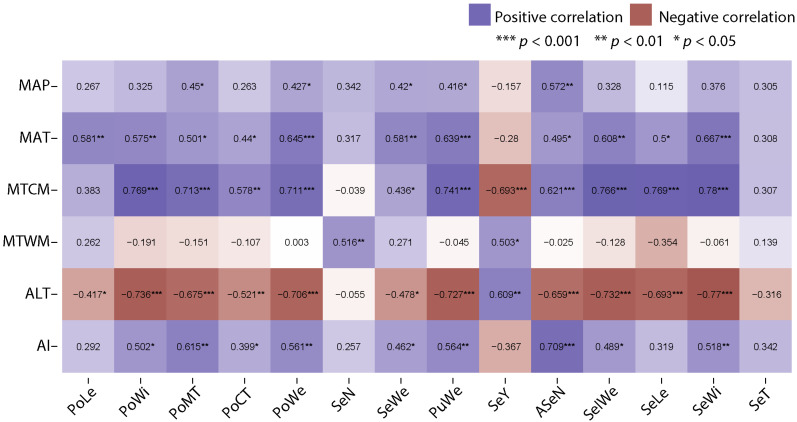
Correlation analysis between carob traits and environmental variables. Abbreviations for pod and seed traits are given in Table 1, and the abbreviations for environmental factors are given in the Section 4.

Correlation analysis indicates that carob morphological traits were significantly associated with the environmental conditions within the range of the species distribution in Morocco (Figure 3). Except for SeN and SeT, all traits were negatively correlated with altitude. In contrast, all traits, except for PoLe, SeN, SeY, SeLe, and SeT, were positively correlated with an aridity index (AI). Additionally, carob traits showed a positive correlation with mean annual temperature (MAT), except for SeN, SeY, and SeT. Seed number (SeN) and seed yield (SeY) were the only traits that had a positive correlation with the mean temperature of the warmest month (MTWM). Weak and positive correlations were observed between mean annual precipitation (MAP) and PoMT, PoWe, SeWe, and PuWe (Figure 3). Of note, seed number (SeN) was found significantly and positively correlated only with MTWM. Moreover, seed yield (SeY) was positively correlated with altitude (ALT) and MTWM and negatively correlated with the mean temperature of the coldest month (MTCM). These findings were further confirmed by the regression analysis (Supplementary Figure S3), which demonstrated a significant and positive relationship between SeY and altitude (R² = 0.38, *p* = 0.0009) and MTWM (R² = 0.27, *p* = 0.007), and a negative relationship with MTCM (R² = 0.50, *p* < 0.0001).

**Figure 4:**
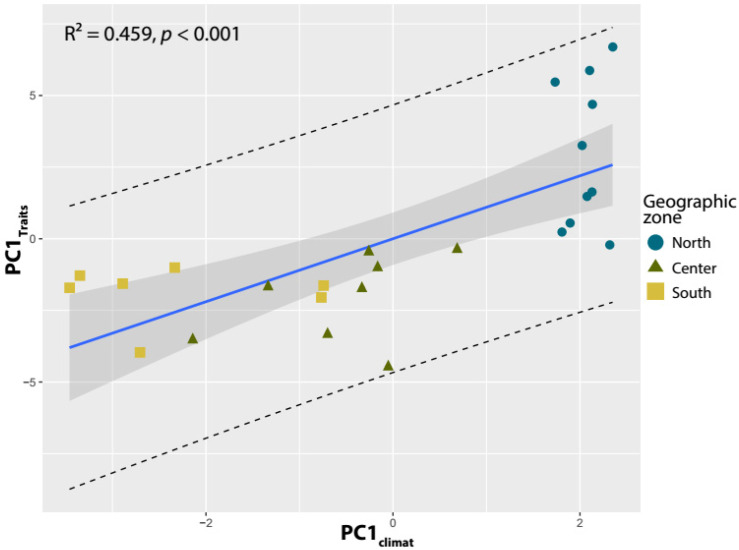
Linear regression model of variation in the slope of fitted lines from regression analysis relating environmental gradients (PC1_Climat_) and carob traits (PC1_Traits_). The transparent line represents the confidence interval for the mean (95%). The dashed line represents the confidence interval for an observation (95%).

The PCA analysis on the 25 carob populations studied using environmental variables indicated that the first PCA axis (hereafter PC1_Climat_), which captured 63.78% of the total variation, was a representative gradient of aridity in the study area, ranging from wet conditions in the north to dry conditions in the south. After performing the regression analysis significant and positive relationship (R² = 0.459, *p* < 0.001) was found between PC1_Climat_ and PC1_Traits,_ which accounted for 65.17% of the total variation in pod and seed traits (Figure 4). These results suggest that as aridity increases from wet to dry conditions, there is a decrease in pod size and dimensions, seed number, and size, while seed yield increases.

## 3. Discussion

There exists a longstanding tradition of investigating the characteristics of plant phenotypes, commonly known as traits, that govern the way in which plants respond to environmental factors [44,45]. Functional traits, encompassing morphological, physiological, and phenological features measured at the individual level, play a crucial role in determining plant performance and indirectly influencing plant fitness [46]. Researchers have extensively employed functional traits as descriptors to capture fundamental trade-offs that define species’ ecological roles and functions [47]. Consequently, a diverse array of adaptive functional strategies in plants has been identified across environmental gradients [2,48,49]. These strategies have primarily been inferred through the assessment of trait variability within and among species, taking into consideration variations in environmental conditions [46,48]. In order to gain insight into whether pod and seed characteristics are linked to a species’ ability to tolerate stressful conditions, it is essential to comprehend the differences in morphological traits of the carob tree along environmental gradients, notably aridity. This understanding could prove valuable in incorporating selection criteria for drought resistance within breeding programs.

### 3.1. Variability in Pod and Seed Traits across the Studied Populations

The study investigated various traits related to carob pods and seeds, and the findings reveal significant differences among the populations (Table 1). The carob tree populations examined in this study exhibited notable variations in all the assessed pod and seed traits, demonstrating a considerable magnitude of variation (e.g., CV%). For instance, the range of pod weight across the carob populations was found to be quite wide, ranging from 15.30 ± 3.83 cm (in the Afertane population) to 3.98 ± 1.35 cm (in the Ait Daoued population). Similarly, seed weight showed considerable variation, ranging from 2.58 ± 0.56 g (in the Afertane population) to 0.75 ± 0.33 g (in the Ait Daoued population). Moreover, the results indicated a substantial amplitude of variability for both pod and seed weights, with coefficient of variation (CV%) values of 45.03% and 35.11%, respectively. The F-ratio values (211.55 for pod weight and 110.82 for seed weight) further emphasize the significant differences observed among the populations. The study also examined seed yield, which showed significant variability with a CV of 30.25% and an F-ratio of 99.26. This indicates that the carob populations studied exhibited diverse seed yield potentials, with values ranging from 32.85 ± 6.46% (in the Arbaa Tigdouin population) to 16.15 ± 4.54% (in the Imi NTlit population). These findings indicate a high level of phenotypic variability and substantial intraspecific variation within the carob tree species [22,50,51]. Our results align with previous studies that have also highlighted distinct phenotypes in carob pods and seeds obtained from different populations [26,32,34]. The discerned disparities in our observations underscore the presence of substantial variability in the investigated traits, implicating a complex interplay of factors encompassing genetic diversity, environmental conditions, and geographical distribution, as posited by prior research [19,22]. Our investigation elucidates the profound influence of both intrinsic and extrinsic factors on the expression and magnitude of trait variability [52]. The carob tree, exemplified by remarkable genetic diversity [19], interacts intricately with the surrounding environmental milieu within our sampled region. This dynamic interplay likely accounts for the discernible disparities observed in pod and seed traits, although further research is warranted to provide a comprehensive explanation. It is paramount to recognize that plants, by their sessile nature, are perpetually subjected to an array of extrinsic sources of variability, notably the flux in environmental conditions. This very dynamism is mirrored in the variability witnessed in the expressed phenotypes [53]. Intriguingly, the variability in trait expression across the studied populations is likely a culmination of multifaceted influences encompassing genotype, phenotypic plasticity [39,53], and the historical climatic characteristics unique to each population. Moreover, our findings pertaining to the fluctuation in seed and pod traits, encompassing dimensions, bear significant implications for the realms of agriculture and food industries. These variations have the potential to exert a substantial impact on the economic value and utility of carob crops, as previously discussed [32]. Consequently, disparities in seed yield assume relevance in the context of agricultural practices and the ongoing endeavors aimed at crop enhancement [54]. This differential seed yield could serve as a valuable criterion for the selection of high-yielding varieties [25]. In effect, the pursuit of genotypes boasting enhanced seed yield and substantial fruit size holds the promise of identifying cultivated varieties rich in nutritional content and composition, encompassing elevated levels of phenolic compounds and vitamins [55].

### 3.2. Impact of the Geographic Origin on the Expression of Carob Pod and Seed Traits

The results showed significant differences in mean values of traits per geographic zone, dividing the populations into North, Center, and South regions (Supplementary Table S1). The results indicated that pod length (PoLe), pod central thickness (PoCT), aborted seed number (ASeN), and seed width (SeWi) showed similar variation between the Center and South regions. However, significant differences were found for pod width (PoWi), pod marginal thickness (PoMT), pod weight (PoWe), seed weight (SeWe), pulp weight (PuWe), seed yield (SeY), seed individual weight (SeIWe), seed length (SeLe), and seed thickness (SeT) among the carob populations from the three geographic zones. Additionally, the North and Center regions were found to be similar in terms of seed number (SeN). The observed variability in pod and seed traits suggests that carob populations respond differently to the environmental conditions present within their distribution areas [10,56]. Moreover, the significant relationship found between trait variability and geographic distance (Supplementary Table S2) confirms the influence of environmental conditions on the studied carob traits. It has been demonstrated that heterogeneous and changing environmental conditions can significantly impact the expression and magnitude of trait variability [9,57]. Furthermore, the results of the principal component analysis (PCA) indicate contrasting patterns of variability among carob populations from different regions, particularly between populations from the north and those from the central and southern regions (Figure 2). Multiple comparison tests also reveal that most of the assessed traits exhibit significant differences based on their geographic origins (Supplementary Table S1). The LDA also showed that carob trees from the North region were distinctly separated from those in the Center and South regions (Supplementary Figure S1), indicating a significant divergence in trait patterns between the Northern populations and the populations from the other two regions. The distinct genetic structure observed in Moroccan carob populations, varying from north to south [19], could influence the phenotypic variability of the investigated carob traits. Collectively, these findings, along with the presence of aridity gradients in our sampling area, likely contribute to shaping the phenotypic variability of pod and seed traits in carob populations. However, it is worth noting that several studies have reported significant differences in pod and seed morphological traits among different carob cultivars [25,54,58].

### 3.3. Structure and Extent of Intraspecific Trait Variability in Carob Tree

Taking into account the hierarchical levels examined in this study (Figure 6), our findings reveal a substantial extent of variability in both pod and seed traits, indicating significant intraspecific variation at local and regional scales (Figure 1). Indeed, variation in functional traits occurs at both the inter- and intra-specific levels and is influenced by evolutionary history and environmental factors [46,59]. Recent research suggests that intraspecific trait variation (ITV) contributes significantly to the overall trait variation, with notable implications for ecological and evolutionary processes [60,61]. Global meta-analyses have demonstrated the significant effect of environmental gradients in shaping the extent and structure of this variability [40,41]. Therefore, considering intraspecific trait variation in trait-based studies can greatly enhance our understanding of how individual plant species respond to environmental gradients, particularly those distributed across wide-ranging environmental conditions [8,22,40,62]. In this study, focusing on the remarkable Mediterranean Ceratonia siliqua tree, we found substantial intraspecific variation in pod and seed-related traits. For example, pod weight exhibited a higher proportion of variance at the inter-geographic level, accounting for 36.55% of the total variability, while seed thickness displayed significant variability at the intra-individual level, accounting for 45% of the total variability (Figure 1). These findings indicate that some traits respond to the variability of environmental conditions acting on a large scale, such as aridity, while others respond to local environmental conditions [8,22]. Furthermore, our results suggest that C. siliqua demonstrates distinct functional responses to the heterogeneous environmental conditions present at both local (e.g., individual tree) and regional (e.g., geographic zone) scales [59,63]. The considerable variability observed at the local scale of individual trees may be attributed to the high genetic diversity expressed in carob populations [19], while inter-geographic variability could be influenced by significant spatial and temporal environmental changes occurring within the distribution area of carob in Morocco [22]. As a result, our findings provide new evidence of pod and seed trait variability within and between carob populations along environmental gradients, contributing to the ongoing efforts in trait-based ecology [46]. Considering these significant findings, the present study demonstrates that trait variation within carob individuals is not negligible and exhibits structured patterns depending on the sampling scales and the intraspecific dimension along environmental gradients. This highlights the important role of phenotypic plasticity in response to aridity gradients.

### 3.4. Carob Pod and Seed Trait Variations and Environmental Variables

The variation observed in seed shape traits appears to be driven by variations in environmental variables, notably climatic variables and altitude. The results demonstrated significant associations between various traits and specific environmental variables (Figure 3). Altitude showed a negative correlation with all traits, except for seed number (SeN) and seed thickness (SeT). This implies that as the altitude increases, carob trees tend to exhibit reduced pod and seed sizes and other related morphological traits. The aridity index (AI) displayed a positive correlation with most traits, except for pod length (PoLe), seed number (SeN), seed yield (SeY), seed length (SeLe), and seed thickness (SeT). This indicates that carob traits tend to increase in response to humid conditions, with carob trees exhibiting larger pod and seed sizes and other related morphological features in areas with low aridity. These findings are in agreement with the ‘energy constraints’ hypothesis [64], assuming that morphological traits can be negatively correlated with elevation, due to low development in high elevations and low temperatures. Seed number (SeN) and seed yield (SeY) were the only traits that had a positive correlation with the mean temperature of the warmest month (MTWM). This implies that the number of seeds produced by carob trees and seed yield both increase with higher temperatures during the warmest month. Interestingly, weak and positive correlations were observed between mean annual precipitation (MAP) and pod marginal thickness (PoMT), pod weight (PoWe), seed weight (SeWe), and pulp weight (PuWe). This suggests that certain traits show a response to precipitation levels, potentially reflecting the influence of water availability on these morphological characteristics. Previous findings showed that variation in seed [22,65,66] and pod [27,34] traits could be driven by geographical or environmental variables. Moreover, the regression analysis (Figure 4) revealed a positive and significant relationship (R² = 0.459, *p* < 0.001) between PC1_Climat_ and PC1_Traits_, which accounted for 65.17% of the total variation in pod and seed traits. This indicates that as aridity increases, moving from wet to dry conditions, there is a decrease in pod size and dimensions, as well as seed number and size. However, there is an increase in seed yield. Consequently, the observed variability in seed and pod traits related to environmental conditions could be explained by the combined effect of climate variability, water, and nutrient availability [8,67,68]. Our results highlight significant associations between carob morphological traits and environmental conditions within the species distribution range in Morocco. Altitude, aridity index (AI), and mean annual temperature (MAT) were identified as important factors influencing the variation in pod and seed traits. The PCA analysis further confirmed the influence of aridity, showing a gradient from wet to dry conditions, and its relationship with changes in pod size, seed number, and size, as well as seed yield. Understanding these correlations is crucial for gaining insights into the ecological adaptation and response of carob trees to environmental conditions. This knowledge is valuable for conservation efforts, understanding species responses to climate change, and guiding management practices for carob populations.

## 4. Materials and Methods

### 4.1. Study Area and Field Sampling

**Figure 5:**
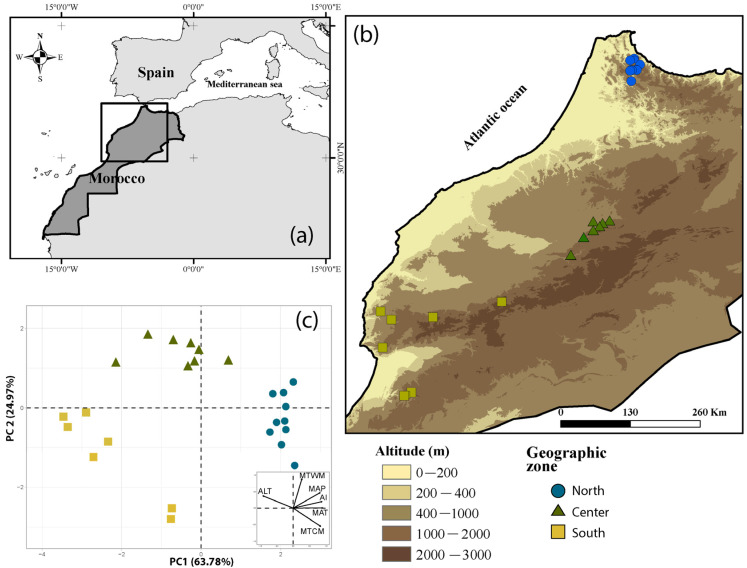
Localization of the study area (**a**) and distribution of the sampled populations according to altitude (**b**) and climatic variables (**c**).

The study took place in Morocco along a latitudinal transect that covers a wide range of environmental conditions across different bioclimatic zones, including sub-humid and semi-arid regions [22,69]. Twenty-five populations of spontaneous carob trees growing under natural conditions were investigated, distributed along a latitudinal gradient from the north to the south region (see Figure 5A–C and Table 2). Each sampling site was defined using six environmental variables: the mean annual temperature (MAT, °C), the minimum temperature of the coldest month (MTCM, °C), the maximum temperature of the warmest month (MTWM, °C), and the mean annual precipitation (MAP, mm), extracted from the Worldclim database at a resolution of 30 arc seconds (~1 km2) [70], the global aridity index (AI, unitless) extracted from CGIAR Global Aridity database [71], and the altitude (ALT, m) recorded using a GPS device (Garmin, GPSmap62, Garmin Ltd., Kansas City, MO, USA).

**Figure 6:**
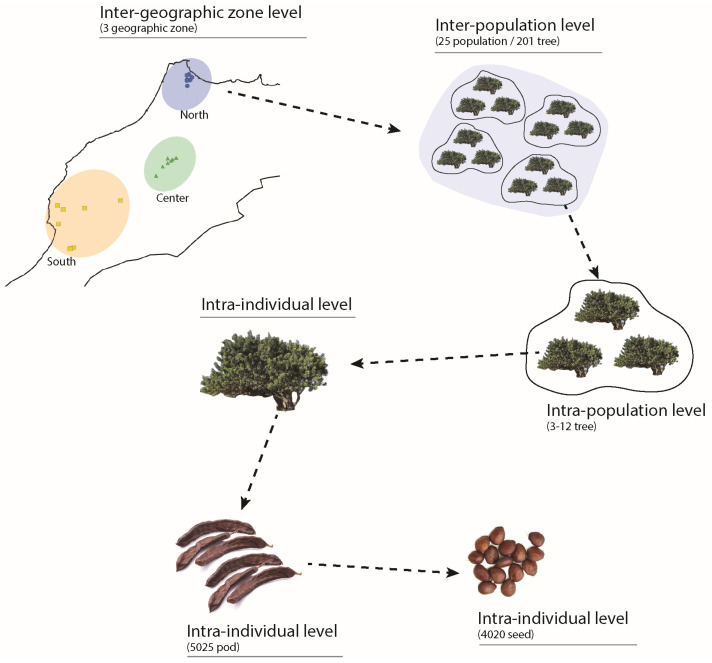
Hierarchical sampling design of the studied carob populations of Morocco.

A total of 201 mature and healthy carob trees were sampled from the investigated populations (Table 2). At each population, trees were randomly selected at a distance of at least 10 to 30 m from each other to capture a range of genetic variations. From each tree, 1 kg of healthy and mature pods was harvested from the four cardinal directions (north, south, east, and west), and 25 pods were randomly selected from the harvest. From the selected pods, 20 seeds were randomly chosen for morphometric analysis, resulting in a total of 5025 pods and 4020 seeds being sampled and measured. This sampling procedure allowed for a four-level hierarchical model, with each level representing a spatial and organizational scale (Figure 6): (1) inter-geographic zone level between different geographic sampling zones (North, Center, and South), (2) inter-population level between different populations, (3) intra-population level between different trees of the same population, and (4) the intra-individual level between different pods and seeds of the same tree (Figure 6).

### 4.2. Morphological Traits Measurements

Fourteen morphological traits were measured, including ten traits related to the pod and four related to the seed (Table 1). The pod traits included pod length (PoLe), pod width (PoWi), pod marginal thickness (PoMT), pod central thickness (PoCT), pod weight (PoWe), seed number (SeN), seed weight (SeWe), pulp weight (PuWe), seed yield (SeY), and aborted seed number (ASeN). The four seed morphometrics measures were seed individual weight (SeIWe), seed length (SeLe), seed width (SeWi), and seed thickness (SeT). A Vernier caliper was used to measure morphological traits, while a precision balance was used to measure the weight.

### 4.3. Statistical Analysis

All statistical analyses were conducted using R software v. 4.0.5 [72]. Descriptive analyses, such as mean and standard deviation, were used to identify general trends in pod and seed-related traits. The coefficient of variation was calculated as CV(%) = standard deviation_trait_/mean_trait_ × 100 [73] to evaluate the range of trait variability. To evaluate the differences in individual pod and seed traits among populations and geographic zones, one-way ANOVA was conducted, and the mean values were compared using the Tukey HSD post hoc test with a significance level of *p* = 0.05, using the multicomp [74] and agricolae R packages [75]. ANOVA assumptions were tested for normality and homoscedasticity using Shapiro–Wilk and Levene tests, respectively. Mantel tests with 999 permutations were performed with the *Mantel* function in the vegan package [76] to determine the relationship between geographic distance and variation in pod and seed traits among carob populations. Trait distances were calculated as the Euclidean distance using the *dis* function [77]. Principal component analysis (PCA) was performed to compare inter-population trait values and to assess the possible effect of environmental variables on the studied populations using the FactoMiner package in R software [78]. A linear discriminant analysis (LDA) was performed to discriminate carob trees based on their geographic origin (e.g., North, Center, and South) using the MASS package [79]. The LDA analysis was conducted using mean values per individual tree. To quantify the variance of traits across the studied hierarchical levels (see Figure 6), a variance decomposition procedure was applied [11,22,51]. The *lme* function in the nlme package [80] was used to fit a general linear model using the restricted maximum likelihood method (RMEL) across the studied levels, and then the *varcomp* function in the ape package [81] was used to extract the variance expressed at each level. To assess the correlation between carob traits and environmental variables, Pearson correlations were conducted using the *cor* function. To examine the relationships between carob morphological traits and environmental gradients, linear regression models were used and R^2^ was used to evaluate the explanatory power of the regression models using the *lm* function.

## 5. Conclusions

The present comprehensive study has provided profound insights into the intricate variations and associations of carob morphological traits across various ecological scales and their intricate relationship with the prevailing environmental conditions within the expansive species distribution range across Morocco. The results of our investigation have unveiled noteworthy disparities in morphological traits among carob populations at different ecological levels. This variation was observed not only within individual trees but also between geographic zones and among distinct populations. It is worth noting that pod-related traits exhibited a more pronounced variability compared to seed traits. This discrepancy underscores the combined influence of genetic diversity, as previously demonstrated for Moroccan carob and environmental factors on the observed trait variations. Furthermore, our study has established robust links between carob morphological traits and specific environmental conditions, signifying the substantial role played by environmental factors in sculpting the morphological characteristics of carob trees in diverse regions. Notably, aridity emerged as a particularly influential determinant. Our findings delineate a discernible aridity gradient, transitioning from relatively wet conditions in the North to progressively drier conditions in the South. This gradient is strongly correlated with shifts in pod size, seed number, seed size, and seed yield. These observations underscore the pivotal role of environmental conditions in driving trait variation across our study area. Moreover, our research has underscored the existence of substantial intraspecific variation in the studied traits, spanning from local to regional ecological scales. This suggests that carob trees exhibit divergent responses to varying environmental conditions encountered within their distribution range in Morocco. This diversity of responses underscores the critical importance of incorporating intraspecific variability into the study and conservation of this remarkable species. Overall, our study significantly enhances our understanding of the ecological adaptation and evolutionary processes at play in carob trees. The observed variations in morphological traits and their correlations with environmental conditions hold significant implications for the development of conservation strategies, assessments of genetic diversity, and management practices within carob populations. Furthermore, these findings shed light on the potential impacts of climate change on carob populations and underscore the necessity of considering environmental factors when researching and conserving plant species within their native habitats. In essence, our study provides a holistic perspective on the intricate interplay between carob morphological traits and environmental conditions, thereby enriching our understanding of the species’ ecology and offering invaluable guidance for future research and conservation endeavors in the face of evolving environmental dynamics.

## Figures and Tables

**Table 1:** Summary statistics of the corresponding pod and seed traits and their measurement units and abbreviations.

Trait	Abbreviation	Units	Mean	S.D	Min	Max	CV (%)	ANOVA *F*
***Pod traits***								
Pod length	PoLe	cm	13.00	2.56	3.8	22.7	19.70	85.58 ^***^
Pod width	PoWi	mm	17.99	2.74	9.15	28.89	15.23	175.79 ^***^
Pod marginal thickness	PoMT	mm	6.55	1.27	3.44	12.98	19.43	132.22 ^***^
Pod central thickness	PoCT	mm	4.53	1.16	1.52	9.76	25.60	56.55 ^***^
Pod weight	PoWe	g	8.68	3.91	1.5	33.53	45.03	211.55 ^***^
Seed number	SeN	number/pod	10.17	2.68	1	18	26.36	77.45 ^***^
Seed weight	SeWe	g	1.81	0.63	0.11	4.18	35.11	110.82 ^***^
Pulp weight	PuWe	g	6.87	3.47	1.21	30.42	50.51	215.70 ^***^
Seed yield	SeY	%	22.40	6.77	3.15	51.70	30.25	99.26 ^***^
Aborted seed number	ASeN	number/pod	0.80	1.13	0	9	141.22	29.69 ^***^
***Seed traits***								
Seed individual weight	SeIW	g	0.18	0.038	0.07	0.4	20.25	127.30 ^***^
Seed length	SeLe	mm	9.07	0.959	5.68	12.08	10.57	76.65 ^***^
Seed width	SeWi	mm	6.80	0.730	3.71	9.83	10.74	72.05 ^***^
Seed thickness	SeT	mm	4.28	0.493	2.12	6.92	11.51	38.26 ^***^

Values labeled with “***” are statistically significant at *p* < 0.001; S.D: standard deviation; Min: minimum values; Max: maximum values; CV (%): coefficient of variation.

**Table 2:** Geographic locations and climate characteristics of the studied populations of *Ceratonia siliqua* L. in Morocco.

Population Name (Tree Number Per Pop.) ^a^	Geo. Zone	Latitude (°)	Longitude (°)	ALT (m)	MAP (mm)	MAT (°C)	MTCM (°C)	MTWM (°C)	AI
Iaasfa (7)	North	35.456	−5.288	418	727	17.1	5.9	30.5	0.601
Toughza (10)	North	35.440	−5.280	264	682	17.7	6.7	30.8	0.561
Amtel (10)	North	35.425	−5.357	389	789	17.3	5.8	31.0	0.65
Afertane (10)	North	35.353	−5.188	31	588	18.5	7.8	31.2	0.499
Zaouia (10)	North	35.283	−5.347	546	868	16.6	4.4	31.7	0.694
Ikhoujja (10)	North	35.269	−5.258	223	704	17.8	6.1	32.0	0.573
Taghzoute (10)	North	35.262	−5.244	326	732	17.4	5.6	31.8	0.586
Kebbache (10)	North	35.256	−5.328	380	820	17.3	5.2	32.0	0.651
Taghbaloute (19)	North	35.249	−5.356	480	876	17.0	4.6	32.1	0.695
Bni Ouella (10)	North	35.076	−5.338	446	914	17.4	4.6	33.2	0.682
Tizim Tazart (10)	Center	32.737	−5.701	886	649	16.4	1.6	35.4	0.435
Sidi Said (6)	Center	32.720	−5.975	686	648	17.8	2.8	37.3	0.409
Ait Taleb (4)	Center	32.691	−5.819	925	705	16.3	1.4	35.8	0.465
Komch (10)	Center	32.638	−5.867	1116	702	15.5	0.3	35.4	0.471
Imheouach (10)	Center	32.573	−5.975	1288	677	14.8	−0.5	35.1	0.46
Tongha (5)	Center	32.455	−6.134	1000	659	16.6	1.2	36.8	0.422
Ouaouizeght (4)	Center	32.154	−6.352	877	519	17.3	2.1	37.0	0.324
Arbaa Tighdouin (10)	Center	31.368	−7.512	1278	447	15.4	−0.6	34.7	0.293
Imi N’Tlit (3)	South	31.203	−9.546	527	326	15.8	5.7	24.8	0.303
Tadart (10)	South	31.109	−8.667	1282	445	13.3	−0.6	28.5	0.347
Ait Daoued (3)	South	31.066	−9.362	1173	414	13.0	1.2	25.2	0.367
Immouzzer Ida Outanane (6)	South	30.598	−9.509	517	289	16.6	5.3	26.4	0.249
Amskassen (6)	South	29.841	−9.026	1478	317	13.8	−0.4	29.1	0.248
Ougougane (5)	South	29.800	−9.117	1133	265	15.6	1.8	29.8	0.213
Talant (12)	South	29.786	−9.148	1409	302	14.0	0.1	28.8	0.223

^a^: the tree number sampled per population is given between parentheses. ALT: Altitude; MAP: mean annual precipitation; MAT: mean annual temperature; MTCM: mean annual temperature of the coldest month; MTWM: maximum temperature of the warmest month; AI: Aridity Index (Low values indicate warmer climate); Geo. Zone: geographic zone.

## Data Availability

The datasets of the current study are available from the corresponding author on reasonable request.

## Supplementary Materials

The following supporting information can be downloaded at: https://www.mdpi.com/article/10.3390/plants12193447/s1, Figure S1: Linear discriminant analysis (LDA) of the studied carob trees according to their geographic origin. Figure S2: Hierarchical cluster analysis of the studied carob populations. Figure S3: Linear regression analysis between seed yield (SeY) and environmental variables. Table S1: One-way ANOVA test and Tukey HSD post hoc results of the studied carob populations according to their geographic origin. Table S2: Mantel test results for the correlation between geographic variations and individual traits of pod and seed. Table S3: Trait loading, eigenvalues and percentage of trait variation explained by the first three principal components (PCs).
