# Supplementary material for: Aridity Gradients Shape Intraspecific Variability of Morphological Traits in Native Ceratonia siliqua L. of Morocco
Source: Plants (Basel). 2023 Sep 30;12(19):3447. doi: 10.3390/plants12193447 (PMC10575131; doi:10.3390/plants12193447)
Supplement: Supplementary file 1 [file plants-12-03447-s001.zip › plants-2622381-supplementary/Table S1.pdf]

Table S1. One-way ANOVA test and Tukey HSD *post hoc* results of the studied carob populations according to their geographic origin.

	North	Centre	South	ANOVA F
**Pod traits** (ANOVA $F_{25,5025}$)				
PoLe	A	B	B	250.65***
PoWi	A	B	C	1063.52***
PoMT	A	B	C	690.14***
PoCT	A	B	B	175.04***
PoWe	A	B	C	1186.43***
SeN	A	A	B	95.39***
SeWe	A	B	C	427.43***
PuWe	A	B	C	1276.48***
SeY	A	B	C	526.14***
ASeN	A	B	B	205.26***
**Seed traits** (ANOVA $F_{25,4020}$)				
SeIWe	A	B	C	551.05***
SeLe	A	B	C	386.34***
SeWi	A	B	B	338.67***
SeT	A	B	C	44.74***

Values labeled with "***" are statistically significant at $p < 0.001$. Levels connected by same letter are not statistically different at $p < 0.05$ in Tukey HSD *post hoc* tests.
