# Supplementary material for: Aridity Gradients Shape Intraspecific Variability of Morphological Traits in Native Ceratonia siliqua L. of Morocco
Source: Plants (Basel). 2023 Sep 30;12(19):3447. doi: 10.3390/plants12193447 (PMC10575131; doi:10.3390/plants12193447)
Supplement: Supplementary file 1 [file plants-12-03447-s001.zip › plants-2622381-supplementary/Table S2.pdf]

**Table S2.** Mantel test results for the correlation between geographic variations and individual traits of pod and seed.

| Traits | Geographic distance | |
|---|---|---|
| ***Pod traits*** | | |
| PoLe | $p = 0.071$, $R_M = 0.117$ n.s. | |
| PoWi | $p = 0.010$, $R_M = 0.212$ | |
| PoMT | $p = 0.007$, $R_M = 0.237$ | |
| PoCT | $p = 0.065$, $R_M = 0.119$ n.s. | |
| PoWe | $p = 0.002$, $R_M = 0.285$ | |
| SeN | $p = 0.008$, $R_M = 0.220$ | |
| SeWe | $p = 0.005$, $R_M = 0.241$ | $p < 0.001$, $R_M = 0.339$[a] |
| PuWe | $p = 0.002$, $R_M = 0.287$ | |
| SeY | $p = 0.057$, $R_M = 0.124$ n.s. | |
| ASeN | $p < 0.001$, $R_M = 0.380$ | |
| ***Seed traits*** | | |
| SeIWe | $p = 0.02$, $R_M = 0.188$ | |
| SeLe | $p = 0.147$, $R_M = 0.070$ n.s. | |
| SeWi | $p = 0.011$, $R_M = 0.193$ | |
| SeT | $p = 0.084$, $R_M = 0.093$ n.s. | |

$R_M$ Mantel statistics; n.s. non-significant; [a] Mantel test using all traits.
