# Supplementary material for: Aridity Gradients Shape Intraspecific Variability of Morphological Traits in Native Ceratonia siliqua L. of Morocco
Source: Plants (Basel). 2023 Sep 30;12(19):3447. doi: 10.3390/plants12193447 (PMC10575131; doi:10.3390/plants12193447)
Supplement: Supplementary file 1 [file plants-12-03447-s001.zip › plants-2622381-supplementary/Table S3.pdf]

Table S3. Trait loading, eigenvalues and percentage of trait variation explained by the first three principal component (PCs).

**Traits**	**PC1**	**PC2**	**PC3**
Pod traits			
PoLe	**0.64**	**0.63**	-0.37
PoWi	**0.93**	-0.17	-0.19
PoMT	**0.86**	-0.25	0.36
PoCT	**0.79**	-0.09	0.47
PoWe	**0.98**	0.12	-0.09
SeN	0.35	**0.92**	0.00
SeWe	**0.82**	**0.56**	0.00
PuWe	**0.98**	0.03	-0.10
SeY	**-0.58**	**0.73**	0.20
ASeN	**0.74**	-0.07	-0.24
Seed traits			
SeIWe	**0.97**	-0.09	0.05
SeLe	**0.85**	-0.29	-0.21
SeWi	**0.90**	-0.20	-0.10
SeT	**0.66**	0.16	**0.65**
Eigenvalues	9.12	2.38	1.12
Proportion explained (%)	65.17	17.01	8.02
Cumulative proportion (%)	65.17	82.17	90.20

Values greater than 0.5 are in bold.
