# Supplementary figures and images for: Aridity Gradients Shape Intraspecific Variability of Morphological Traits in Native Ceratonia siliqua L. of Morocco
Source: Plants (Basel). 2023 Sep 30;12(19):3447. doi: 10.3390/plants12193447 (PMC10575131; doi:10.3390/plants12193447)

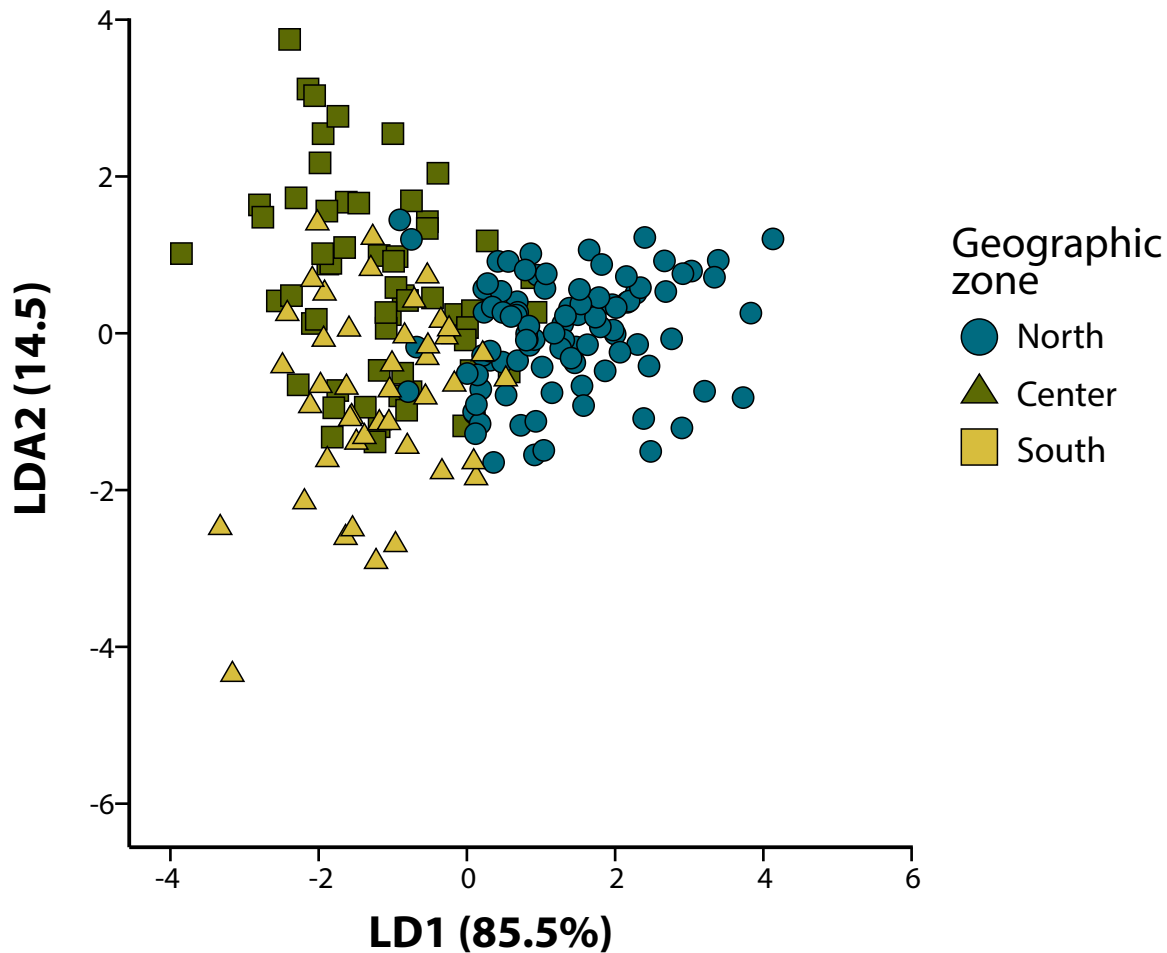

Supplement: Supplementary file 1 [file plants-12-03447-s001.zip › plants-2622381-supplementary/Figure S1.pdf]

# Geographic zone

- North
- Center
- South

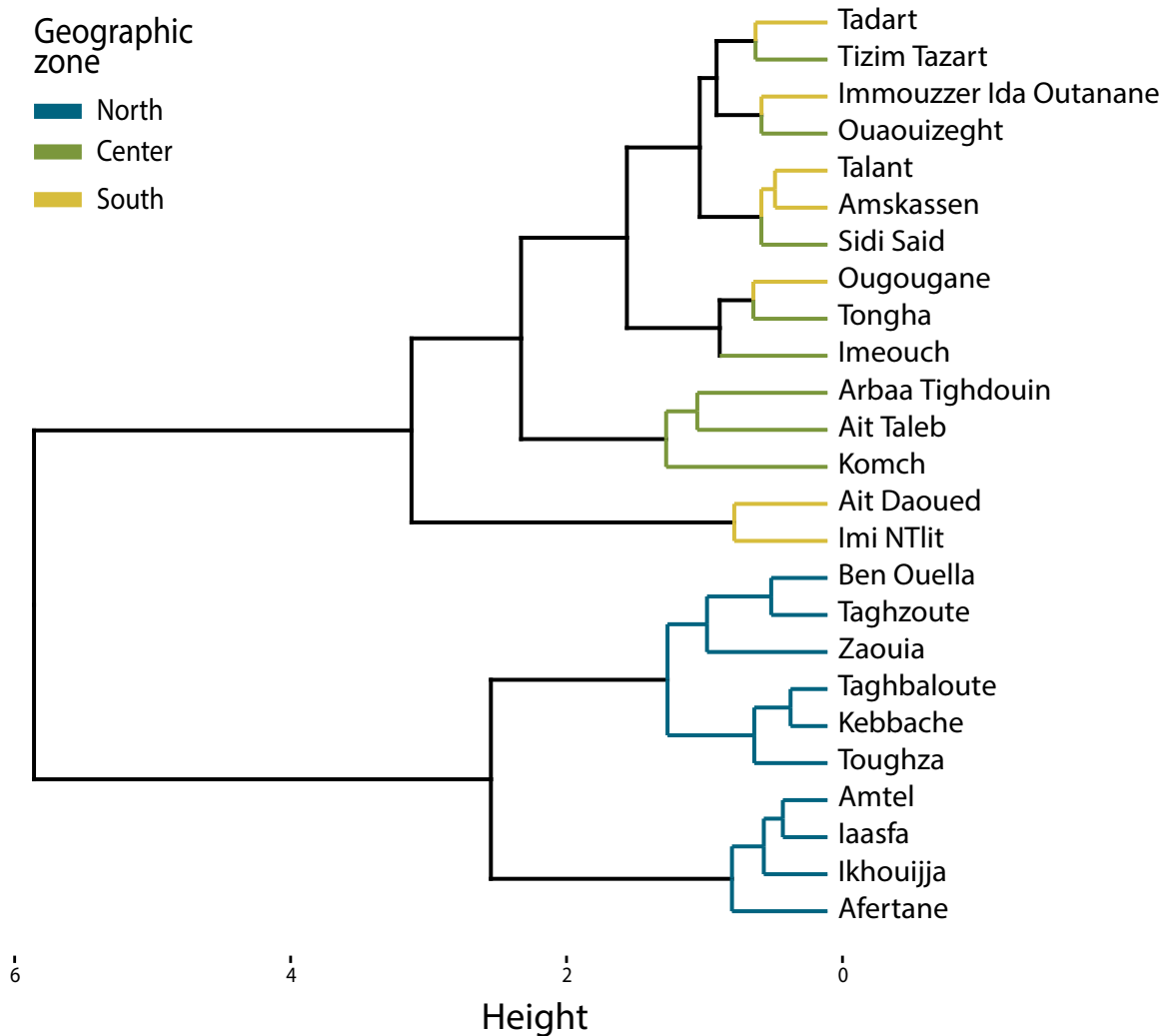

Supplement: Supplementary file 1 [file plants-12-03447-s001.zip › plants-2622381-supplementary/Figure S2.pdf]

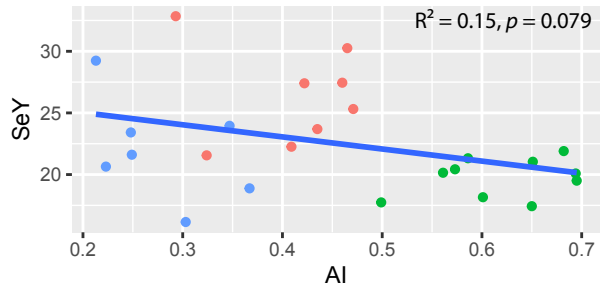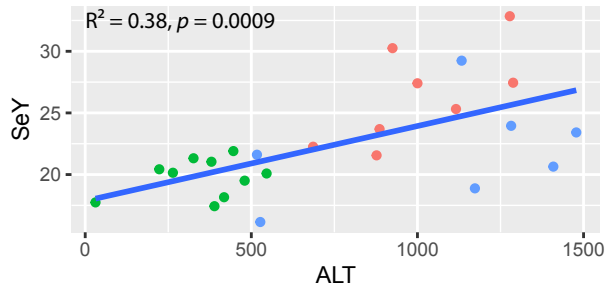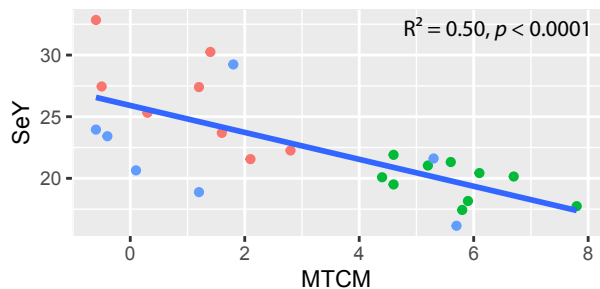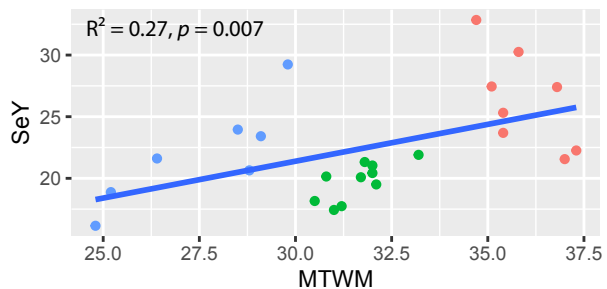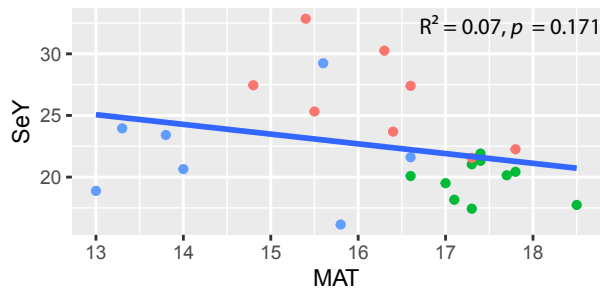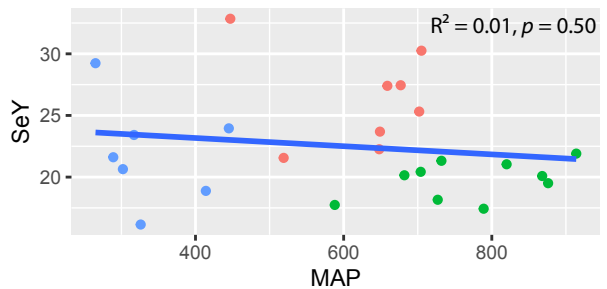

### Geographic zone

- North
- Center
- South

Supplement: Supplementary file 1 [file plants-12-03447-s001.zip › plants-2622381-supplementary/Figure S3.pdf]
